# Stakeholder perceptions affecting the implementation of teleophthalmology

**DOI:** 10.1186/s12913-022-08386-4

**Published:** 2022-08-25

**Authors:** Molly J. E. Snider, April Y. Maa, Arthur C. Guyton, Hannah Park, Kelly J. Hunt, Charlene Pope

**Affiliations:** 1 Cleveland Clinic Cole Eye Institute, 2022 E 105th St I Bldg, Cleveland, OH 44106 USA; 2 Emory University, Atlanta, USA; 3 VISN 7, Regional Telehealth Services, Atlanta Veterans Affairs Medical Center, Atlanta, USA; 4 Atlanta VAMC CVNR, 1670 Clairmont Road, MC150R, Decatur, GA 30033 USA; 5 Private Practice, Roswell, USA; 6 Department of Public Health Sciences, Medical University of South Carolina, Charleston, USA; 7 Ralph H. Johnson Veterans Affairs Medical Center, Charleston, SC; and, Department of Pediatrics, Medical University of South Carolina, Charleston, USA

**Keywords:** Implementation barriers, Implementation science, Stakeholder perceptions, Teleophthalmology, Telehealth, Innovation sustainability

## Abstract

**Purpose:**

Teleophthalmology has become the subject of heightened interest and scrutiny in the wake of the SARS-CoV-2 global pandemic. A streamlined implementation framework becomes increasingly important as demand grows. This study identified obstacles to teleophthalmology implementation through summative content analysis of key stakeholders’ perceptions.

**Design:**

Summative content analysis of transcribed interviews with key stakeholders (including patients, technicians, ophthalmic readers, staff, nurses, and administrators at two teleophthalmology clinic sites).

**Methods:**

Keyword Were counted and compared to examine underlying meaning. Two analysts coded text independently using MAXQDA for summative qualitative content analysis to derive themes and hierarchical relationships as a basis for future refinement of TECS implementation. xMind ZEN was used to map conceptual relationships and overarching themes that emerged to identify perceived facilitators and barriers to implementation

**Results:**

Analysis revealed two themes common to perceptions: (1) benefits of care, and (2) ease of implementation. Perceived benefits included efficiency, accessibility, and earlier intervention in disease course. The quality and quantity of training was heavily weighted in its influence on stakeholders’ commitment to and confidence in the program, as were transparent organizational structure, clear bidirectional communication, and the availability of support staff.

**Conclusion:**

Using a determinant framework of implementation science, this report highlighted potential hindrances to teleophthalmology implementation and offered solutions in order to increase access to screening, improve the quality of care provided, and facilitate sustainability of the innovation.

**Supplementary Information:**

The online version contains supplementary material available at 10.1186/s12913-022-08386-4.

## Contributions to the literature

- Considering the global slant towards telemedicine in the wake of the SARS-CoV-2 pandemic, streamlining program implementation will become a healthcare imperative
- This study identified potential hindrances to teleophthalmology implementation through a multilevel determinant framework of implementation science.
- Factors important to key stakeholders included ongoing hands-on training with formative feedback, dedicated support staff, transparent organizational structure, and clear bidirectional communication

## Background

Telemedicine, or care provided across spatial and/or temporal distance, is often touted as a solution to the critical dilemma posed by a rapidly expanding aging population in countries with inadequate healthcare infrastructures. In ophthalmology, telemedicine is well-validated and widely accepted in diabetic retinopathy screening [1], and more recently has found traction in glaucoma monitoring [2–5], retinopathy of prematurity screening [6, 7], macular degeneration screening [8, 9], and comprehensive ophthalmology [10]. Increasingly inexpensive and ubiquitous technologies, expanding computational power, and surging demand for limited healthcare resources are all pressures that have contributed its popularity. The SARS-CoV-2 pandemic has further accelerated popular use in part due to expanded reimbursement [11], as well as encouragement on the part of governing bodies such as the American Academy of Ophthalmology.

Early data highlights teleophthalmology’s potential to reduce volume burden on clinics, travel burden on patients, and increase access to care for rural, impoverished, and otherwise vulnerable patients [12–14]. Data also suggests teleophthalmology is well-accepted by patients and providers [15–17]. Randomized controlled trials, while limited in number, suggest increased adherence to recommended frequency of diabetic eye exams [18], improved attendance at follow-up visits [19], and no significant difference in visual outcomes or delay to treatment compared to usual care [20]. Pooled sensitivity and specificity for classification of diabetic retinopathy in teleophthalmology were found to be over 80% and 90% (respectively) by a meta-analysis [21]. The United Kingdom successfully integrated store-and-forward telemedicine into its National Health Service Diabetic Eye Screening Programme in 2003. By 2010, Liew reported that diabetic retinopathy had been stripped of its 50-year reign as leading cause of blindness in working-aged adults [22]. By 2018, nearly 83% of eligible individuals were being screened annually [23].

Despite its promise, widespread adoption of teleophthalmology has yet to occur across the public and private sector in North America. This may be attributed more to practical realities of implementation and operation than to its potential utility. Organizational Readiness theory argues that implementation of novel models of healthcare delivery relies on a collective commitment to, and confidence in, the ability to create and sustain organizational change [24–26]. Overcoming resistance and other barriers becomes particularly important as the technology in question reaches the “tipping point,” [27] after which the most influential stakeholder is no longer an early adopter focused on and invested in the technology. Instead, the influence shifts to the public, who is most concerned with practical integration to real life [12]. For this reason, understanding how key stakeholders perceive the innovation is critical to facilitate meaningful implementation and long-term sustainability [28]. This effort to characterize individual determinants of implementation outcomes falls within what is known as a “determinant framework” of implementation science and is a potentially useful antecedent to successfully executed implementation strategies [29]. Using a multilevel determinant framework [29], this study elicited insight from patients, providers, and staff critical to the implementation and early operation of a comprehensive teleophthalmology clinic in an effort to identify remaining unmet needs and eventually facilitate long-term sustainability. At the time of publication, only one other study exploring obstacles perceived by teleophthalmology clinic staff existed [30].

## Methods

### VA comprehensive teleophthalmology clinic operations

One early adopter of teleophthalmology, the Veterans Health Administration (VA) [31–33], established asynchronous diabetic retinopathy screening in primary care clinics in 2006, and expanded its efforts in 2015 to include comprehensive teleophthalmology. Technology-based Eye Care Services (TECS), which now operates out of 40 sites nationally [32–34], is able to prescribe spectacles and screen for glaucoma in addition to diabetic retinopathy through its use of highly-trained ophthalmic technicians. Technicians operate out of primary care clinics and collect screening data including medical and ocular history, best corrected distance and near visual acuity, intraocular pressure (iCare tonometer), pupil check, refractive status (Marco ARK 1S), external photographs, spectral domain OCTs and visual field testing as necessary, and mydriatic, non-stereoscopic, 45 degree bilateral fundus photographs. Technicians upload data into the patient’s electronic health record, where a physician reviews the information remotely. Reading physicians develop their assessment and plan typically within the hour, and may prescribe eyeglasses or refer for in-person exam as needed [10]. Any adult without known eye disease or evidence of acute eye disease is eligible to be seen in TECS.

### Study design

The Atlanta VA Research and Development Department deemed this initiative was quality improvement and quality assurance in nature, therefore Institutional Review Board approval was not required. Information is reported according to Standards for Quality Improvement Reporting Excellence (SQUIRE) guidelines [35]. Participants gave verbal informed consent. Tenets of the Declaration of Helsinki and the Health Insurance Portability and Accountability Act were followed.

Interviewers used a responsive method that relies on rapport-building and probing for understanding [36]. The semi-structured questions appear in Supplementary Materials (Additional file 1: Appendix 1). Rather than a fixed total number in the sample, the sampling frame represents the series of key stakeholders for TECS.

Interviewees included patients, technicians, ophthalmic readers, staff at VA Community-Based Outpatient Clinics (CBOCs), nurses, administrators, and eye clinic providers. A minimum of four participants in each category were sought or until novel content was saturated. Stakeholders were invited to participate via e-mailed letters.

Individual interviews were conducted by a trained qualitative data analyst (A.G.). Interviews were conducted either in person or by video conference using Zoom (a HIPAA-compatible encrypted platform). Interviews were 30–60 min and were audio-recorded with participant consent. Interview recordings were transcribed and de-identified by the VA Informatics, Decision-Enhancement and Analytic Sciences Center (IDEAS) in Salt Lake City, UT. Coders at the VA Health Equity and Rural Outreach Innovation Center (HEROIC) in (Charleston, SC) entered the transcript material into MAXQDA, a qualitative data analysis software [37].

### Data analysis

Interviews were qualitatively analyzed using summative content analysis [38]. This inductive method separates data into analytical codes by counting and comparing keywords followed by interpretation of the underlying context without a pre-established coding frame [39]. Two coders (C.P. and N.P.) reviewed the transcripts and coded the data independently, and discrepancies were resolved by consensus. Analytic categories were delineated and refined using memo writing. Latent content analysis was then applied to the process of interpretation to examine underlying meaning and patterns in the data. Rather than investigator-driven categories and seeing whether data fit into a pre-existing theoretical framework, the categories were derived from the views expressed by the study participants. xMind [40], a concept mapping software, was used to visually map conceptual relationships such that common themes could be identified [41, 42].

## Results

### Interviews

Twenty-nine participants, identified as either patients (P), technicians (T), ophthalmic readers (OR), staff at VA Community-Based Outpatient Clinics (CBOC), nurses (N), administrators (A), and eye service providers (ESP), were interviewed. On average, 10 questions were asked to each group (Additional file 1: Appendix 1). Six veterans, four administrators, three physicians, ten team members, and six technicians.

### Qualitative content analysis

**Fig. 1:**
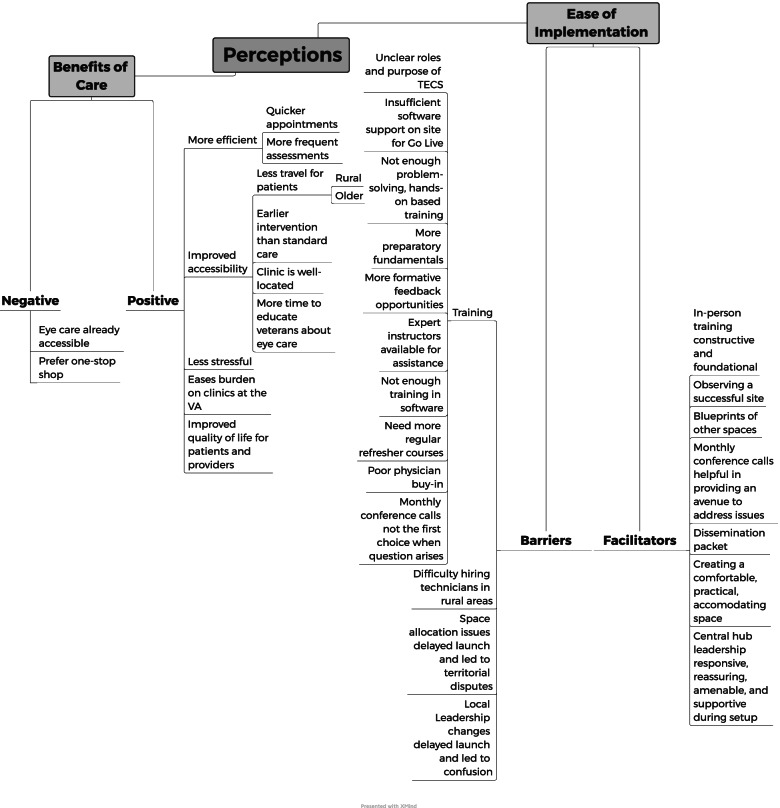
Themes underlying perceptions of TECS implementation, with substantiating coding schema

Perceptions of the implementation process were driven by two major common themes: (1) benefits of care, and (2) ease of implementation. The coding schema used to derive themes and hierarchical relationships is shown in Fig. 1. In Fig. 1, the two common themes underlying stakeholder perceptions can be seen branching from “Perceptions.” From left to right, “Benefits of Care” was further subdivided into “Negative” (or perceived lack of benefits) and “Positive.” Continuing, “Ease of Implementation” was further subdivided into “Barriers (to implementation)” and “Facilitators.” Each branch beyond this point represents analytical codes distilled from all interviews, and substantiating quotes are presented throughout the Results section.

### Perceived benefits of care

Perceived advantages to TECS over usual care included improved efficiency, accessibility, and convenience, especially for rural patients. Staff perceived veterans seeking care at more regular intervals. Veterans enjoyed a reduced travel burden and greater appointment flexibility. Ophthalmic readers observed that TECS implementation led to earlier intervention in the course of their patients’ illnesses. Higher quality of life for patients and staff was a recurrent theme.

*T350010: “The patients absolutely love it. They come back to me instead of going over to the hospital. They don’t have to wait. Patients really appreciate [us] being in their [community]. I've had the head of the department actually come and say we have zero percent wait time for a patient that's having a problem.”*

Those who perceived fewer personal benefits or already had convenient access to eye care preferred the “one-stop shop” of the VA:

*OR350019: “In our program, I don’t think [TECS] made much of an impact because the two sites already have optometry coverage.”*

### Perceived ease of implementation

#### Facilitators of implementation

The in-person training in Atlanta, GA was found to be constructive and uncomplicated. Administrators reported adequate support from Atlanta leadership during implementation, including monthly conference calls to address concerns. A dissemination packet provided as a guide was “unintimidating” and had “very good, basic information.” Patients, technicians, and CBOC staff found the spaces comfortable, charming, and practical. Administrators appreciated the practical assistance provided (e.g., blueprints of other functioning spaces), as this helped to reduce resistance and anxiety.

#### Barriers to implementation

Training was a major theme underlying concerns among individuals spanning all stakeholder groups. Most interviews identified one or more of the following needs: (a) hands-on, in-person training, (b) guidance and formative feedback from an experienced teacher, and (c) protocol to follow when uncertain. Administrators found a need for more guidance when navigating the challe nges of finding clinic space and securing funding. CBOC staff wanted to know more about the myriad roles and responsibilities within their TECS site so that they could help streamline patient flow. Ophthalmic readers, technicians, administrators, and other CBOC staff all voiced a need for easily accessible guidance protocols:

*OR350024: “It's important to have everything very clearly outlined. For example, exactly what clinics to schedule into, how to schedule that, [and do I] overbook? I think having templates for our exam should also be very streamlined.”*

*CBOC350012: “Maybe following somebody who is established in the program and up and running in their own clinic [would help] get a good feel for the patient flow, how things work [so you can understand] why we do [what we do], how we deal with the consults, how the images get transferred into the computer, how the reader actually sees them. That way everything can kind of click when you see it [from all] sides.”*

*A350031: “Having some training videos for the technicians on how to use the equipment, [take] photographs, [perform] different parts of the exam, that would be really helpful.”*

Administrators and CBOC staff identified a need for experienced TECS personnel to be present for the first few days surrounding site launch:

*A350030: “I feel like Atlanta needs to have a dedicated person that travels out and hits the ground [running] at the sites and basically within the course of a day or two can start the entire site, get it up and running, troubleshoot for the first few days of clinic.”*

Some stakeholders faced resistance from other CBOC occupants. Misunderstanding the goals of TECS was reportedly responsible for disputes among other clinics sharing the space and interdepartmental “mistrust.” When TECS work relied on cooperation from unaffiliated parties such as shared CBOC schedulers and check-in clerks, daily tasks became arduous:

*T350010: “When I came here, it was a hostile work environment. [Other CBOC staff] felt like they were being invaded, and it was a strong territorial fight. The MSAs or clerks (who check in patients and set up appointments) [don’t always support me]. The dental program will still remind me that this is their space.”*

Several technicians and CBOC staff felt unheard and undervalued. Despite bringing safety hazards to the attention of their superiors, they saw nothing done. They identified a need for the whole site to periodically convene and discuss issues for all stakeholders:

*T350010: “I don’t think all of the parties were ever brought to the table and informed as to what was happening. It creates a loss of efficiency [and] safety hazards. I have to move the camera out, and it's [a] trip hazard. Although we alerted the safety department, nothing has changed. And I mean I've actually caught two or three patients from falling.”*

## Discussion

As Lorenzi notes, the most effective, beneficial, cost-saving new technology can be “brought to its knees” by stakeholders with a low sense of ownership, confidence or investment in the new system [43]. This study identified potential hindrances to teleophthalmology implementation through summative content analysis using a determinant framework of implementation science. Factors important to stakeholders included ongoing hands-on training with formative feedback, transparent organizational structure, clear bidirectional communication, and dedicated support staff.

Training was brought up extensively throughout interviews, marking the topic as critical to stakeholder investment. Technicians wished for more extensive hands-on training with formative feedback, administrators for clearer blueprints for operations, and physicians for clearer instructions. Ongoing training for all team members was also identified as a particularly important topic by Ramchandran et al. [30] Substantial evidence supports the utility of formative feedback, defined as specific, timely, supportive, information conveyed to the learner in response to an action with the intent to modify the learner’s thinking or behavior, in the development of procedural and motor skills [44]. Administrators whose responsibility included securing funding and space identified the need for “blueprints” for success, or advice from more experienced peers. This group may benefit from structured peer mentoring and support. Physicians wished for more guidance by way of a clear protocol to follow when uncertain. Similarly, administrators expressed need for a “super user” available to assist during site opening. Project managers remaining available during Go-Lives is a common feature of many health technology products.

Miscommunication and insufficient buy-in led to issues ranging from safety hazards to a hostile work environment. Stakeholders felt they could not identify how their role fit into the larger picture, and did not have a good understanding of the full clinical process. Better training, transparent organizational structure, and clear bidirectional communication may address these concerns. Fostering a culture which regularly seeks feedback and acts upon it is especially important to successful implementation; feeling heard and empowered is critical to buy-in [45–47]. Although likely situational, others may still learn from the description of hostile sentiments towards TECS occupants of CBOC space for future planning. Interviews revealed misunderstanding and mistrust of TECS:

*T350010: “The people on the frontlines … they don’t know who you are or what you’re doing or why. Get down to the grassroots…the people that are going to check [patients] in for you…[get] them on board before you start.”*

Reaching out before site launch to let occupants of the incoming space know what the clinic is and how important the current occupants are to the mission of providing vision-preserving care to our vulnerable veteran population may ease misunderstandings.

### Limitations

Limitations of this study include the use of convenience sampling, non-standardized questions across groups, and unequal numbers of participants in each stakeholder group. Similarly, demographic data was not collected thus limiting contextual grounding of our results. In addition, by following summative qualitative methods, the opportunity to perform a mixed methods or quantitative analysis was missed. The hostility felt by CBOC staff was unexpected, thus the authors were unable to secure interviews with non-TECS parties; however, other health professionals sharing CBOC space with TECS stakeholders should be included in future quality improvement research in order to better understand relational obstacles.

## Conclusions

Using a determinant framework of implementation science, this study identified several factors important to stakeholders, including ongoing hands-on training with formative feedback, dedicated support staff, transparent organizational structure, and clear bidirectional communication. Looking forward, meeting these needs has the potential to increase access to screening, improve the quality of care provided, and facilitate sustainability of the innovation.

## Supplementary Information

**Additional file 1.**

## Data Availability

The datasets used and/or analysed during the current study are available from the corresponding author on reasonable request.

## Abbreviations

VA: Veterans Health Administration
TECS: Technology-based Eye Care Services
CBOC: Community-based Outpatient Clinic
SQUIRE: Standards for Quality Improvement Reporting Excellence
