# Supplementary material for: Stakeholder perceptions affecting the implementation of teleophthalmology
Source: BMC Health Serv Res. 2022 Aug 25;22:1086. doi: 10.1186/s12913-022-08386-4 (PMC9403222; doi:10.1186/s12913-022-08386-4)
Supplement: Supplementary file 1 — Additional file 1. [file 12913_2022_8386_MOESM1_ESM.docx]

**Supplementary Files**

Appendix 1: TECS Implementation Study Interview Questions:

Patients:

We are interested in your experiences with eye exams. I would like to ask you some questions so that we can try to continually improve our services.

1. How often do you have an eye exam?
   1. Where do you go to have your eye exam?
      1. *(if response above is TECS)* Where did you go before this service was available?
   2. What (prevents, prevented) you from having regular eye exams?
2. I would like to ask you about the Technology-based Eye Care Services program that we call TECS that is available at your Community Based Outpatient Clinic (CBOC). TECS screens for common eye problems so that you can receive your exam for your glasses at your CBOC. How (has, will) having this program affect how often you have an eye exam?
3. What has your experience been with the TECS program?
   1. Please share more about that…
   2. What would you tell other Veterans about the TECS program?
   3. If you have had experience with the TECS program, what would you like to change?
4. When you received your screening, you were advised that you needed follow up at your medical center?
   1. How did you feel about the notification and scheduling of your follow up?
5. *[Blairsville pt.]* The TECS program was available at your CBOC and was temporarily closed due to personnel availability; it is now reopened. How do you feel about this?
6. How do you feel TECS has helped you learn more about preventive care?

TECS Technicians:

The purpose of this implementation work is to support internal evaluation efforts by the TECS program necessary for ORH and Atlanta VA Health Care Systems QA/QI reporting and program assessment. Additionally, we want to continually improve the experience for patients, technicians, and providers.

1. What was your previous clinical experience prior to becoming a TECS technician?
2. We would like to know about your training experience. Please tell us about the training your received to learn the Technology-based Eye Care Services protocol, and to work as a TECS technician.

a) How do you feel about the training you received to implement the protocol?

b) What recommendations do you have for training new technicians?

1. TECS is designed to work in a small space of 120 sq. ft., what was it like for you setting up your TECS area or space?
2. Tell me about the challenges of the space you have in your CBOC?
3. What do you find is positive about your space?
4. To improve the TECS visit experience for both the patient and the technician, and to ensure the standardization of the protocol implementation, what recommendations do you have regarding in-services or periodic refresher trainings?
5. How do you feel the TECS program has been received by your CBOC?
6. Please give me an example of how primary care or your CBOC helps you in letting the Veterans know that the TECS program is available?
7. How has the staff indicated to you that TECS is beneficial to patient access and care at your CBOC?
8. What recommendations would you offer to new TECS sites?
9. How do you educate the patient about the importance of regular eye screening, and keeping any follow-up appointment that might be scheduled following the TECS screening they receive from you?
   1. During the TECS screening, when you reach a protocol stopping point that requires you to contact the reading physician, how do you communicate to the patient the need to discontinue the screening?
10. Please share any additional thoughts or information that you would like TECS to know...

TECS Physician Readers:

The purpose of this implementation work is to support internal evaluation efforts by the TECS program necessary for ORH and Atlanta VA Health Care Systems QA/QI reporting and program assessment. Additionally, we want to continually improve the experience for patients, technicians, and providers.

1. As a physician reader that also provides traditional eye care, how do you feel about Technology-based Eye Care Services screening in comparison to the service you provide to patients during a face-to-face exam?
2. How do you feel that TECS has affected Veterans access to eye care?
3. How do you feel about the education the technician provides to the Veteran during a TECS screening?
4. What feedback have you received from Veterans when you contact them regarding following up on your findings?
5. What are your thoughts regarding the peer review process?
6. What training did you receive in preparation for being a TECS reader? What training would you recommend?
7. What recommendations would you make to a physician that will be reading TECS?
8. Please share any additional thoughts or information that you would like TECS to know...

CBOC Personnel:

The purpose of this implementation work is to support internal evaluation efforts by the TECS program necessary for ORH and Atlanta VA Health Care Systems QA/QI reporting and program assessment. Additionally, we want to continually improve the experience for patients, technicians, and providers.

- Primary care providers:

1. How has having the TECS program available affected your CBOC?
2. What have Veterans shared with you regarding having TECS in the clinic?
3. What do you share with Veterans regarding TECS?
4. How have you assisted Veterans with learning more about TECS?
5. *[Blairsville only]* The TECS program was available at your CBOC and was closed due personnel availability. How did this impact the Veterans who you serve?
   1. Now that the TECS screening again available, how does this impact the Veterans you serve?
6. What would be your recommendations for new TECS sites that are implementing the program in their CBOCs?
7. How do you feel TECS helps Veterans learn more about preventive care?
8. Please share any additional thoughts or information that you would like TECS to know...

- Staff/ Nurse:

1. How has having the TECS program available affected your CBOC?
2. What have Veterans shared with you regarding having TECS in the clinic?
3. What do you share with veterans regarding TECS?
4. How have you assisted Veterans with scheduling a TECS visit?
5. *[Blairsville only]* The TECS program was available at your CBOC and was closed due personnel availability. How did this impact the Veterans who you serve?
6. Now that the TECS screening again available, how does this impact the Veterans you serve?
7. What would be your recommendations for new TECS sites that are implementing the program in their CBOCs?
8. Please share any additional thoughts or information that you would like TECS to know...

- CBOC Leadership/Med Center Leadership:

1. How has having the TECS program available affected your CBOC?

a) TECS is designed to operate in a small space. How do you feel about the space utilized by TECS at your facility?

1. What feedback have you received from Veterans regarding TECS at your CBOC?
2. Please share some examples of staff feedback…
3. What suggestions do you have for introducing TECS to the providers and staff at your clinic?
4. How would you introduce the availability of TECS to Veterans at your clinic?
5. What recommendations would you have for CBOCs that will be implementing the TECS program at their clinic in the future?
6. Please share any additional thoughts or information that you would like TECS to know...

Eye Providers (AVAMC):

The purpose of this implementation work is to support internal evaluation efforts by the TECS program necessary for ORH and Atlanta VA Health Care Systems QA/QI reporting and program assessment. Additionally, we want to continually improve the experience for patients, technicians, and providers.

1. How has Technology-based Eye Care Services at the CBOCs affected access to eye care service at this facility?
2. What feedback have you received from Veterans that have been referred to the eye clinic for additional follow up?
3. What understanding did the patients have regarding the reason for the referral?
4. How has the TECS program affected patient education regarding regular eye screenings?
5. Please share any additional thoughts or information that you would like TECS to know...
